# Supplementary material for: Geodemographic Area Classification and Association with Mortality: An Ecological Study of Small Areas of Cyprus
Source: Int J Environ Res Public Health. 2019 Aug 15;16(16):2927. doi: 10.3390/ijerph16162927 (PMC6720657; doi:10.3390/ijerph16162927)
Supplement: Supplementary file 1 [file ijerph-16-02927-s001.pdf]

Supplementary Material

Table S1. Posterior median and 95% credible intervals for the percentage of spatially structured variability to the total variability in each indicator.

Indicator	Median (95% Credible Intervals)
Age 0–14	84.0 (70.2, 100.0)
Age over 65	81.2 (69.9, 90.3)
Non Cypriot population	60.7 (38.7, 80.2)
Married population	44.8 (25.1, 64.4)
Divorced population	0.2 (0.1, 4.3)
Single-person households	52.4 (31.9, 71.1)
Single-parent households	30.6 (7.4, 54.9)
Six and over members	78.9 (55.9, 93.5)
Not owner occupied	66.3 (43.1, 83.4)
Privately renting	52.6 (32.3, 70.3)
Multi dwelling house	68.8 (53.1, 81.2)
Secondary/Seasonal	100.0 (99.9, 100.0)
Vacant/For demolition	41.3 (7.0, 67.3)
Construction after 2000	68.0 (42.4, 86.6)
Education	84.9 (74.5, 93.1)
No PC in household	78.5 (66.2, 88.4)
Unemployment	3.7 (0.0, 43.0)
Agricultural workers	100.0 (99.9, 100.0)
Elementary occupations	0.0 (0.0, 33.3)
